# Low pH Exposure During Immunoglobulin G Purification Methods Results in Aggregates That Avidly Bind Fcγ Receptors: Implications for Measuring Fc Dependent Antibody Functions

**DOI:** 10.3389/fimmu.2019.02415

**Published:** 2019-10-11

**Authors:** Ester Lopez, Nichollas E. Scott, Bruce D. Wines, P. Mark Hogarth, Adam K. Wheatley, Stephen J. Kent, Amy W. Chung

**Affiliations:** ^1^Department of Microbiology and Immunology, Peter Doherty Institute for Infection and Immunity, University of Melbourne, Parkville, VIC, Australia; ^2^Immune Therapies Group, Burnet Institute, Melbourne, VIC, Australia; ^3^Department of Immunology and Pathology, Monash University, Melbourne, VIC, Australia; ^4^Department of Clinical Pathology, The University of Melbourne, Melbourne, VIC, Australia; ^5^Infectious Diseases Department, Melbourne Sexual Health Centre, Central Clinical School, Alfred Health, Monash University, Melbourne, VIC, Australia; ^6^ARC Centre of Excellence in Convergent Bio-Nano Science and Technology, University of Melbourne, Parkville, VIC, Australia

**Keywords:** Fcγ receptors, IgG purification, protein G, melon gel, antibody, antibody dependent cellular phagocytosis (ADCP), Fc functions

## Abstract

Evaluating the biophysical and functional nature of IgG is key to defining correlates of protection in infectious disease, and autoimmunity research cohorts, as well as vaccine efficacy trials. These studies often require small quantities of IgG to be purified from plasma for downstream analysis with high throughput immunoaffinity formats which elute IgG at low-pH, such as Protein G and Protein A. Herein we sought to compare Protein G purification of IgG with an immunoaffinity method which elutes at physiological pH (Melon Gel). Critical factors impacting Fc functionality with the potential to significantly influence FcγR binding, such as IgG subclass distribution, *N*-glycosylation, aggregation, and IgG conformational changes were investigated and compared. We observed that transient exposure of IgG to the low-pH elution buffer, used during the Protein G purification process, artificially enhanced recognition of Fcγ Receptors (FcγRs) as demonstrated by Surface Plasmon Resonance (SPR), FcγR dimer ELISA, and a functional cell-based assay. Furthermore, low-pH exposed IgG caused conformational changes resulting in increased aggregation and hydrophobicity; factors likely to contribute to the observed enhanced interaction with FcγRs. These results highlight that methods employed to purify IgG can significantly alter FcγR-binding behavior and biological activity and suggest that the IgG purification approach selected may be a previously overlooked factor contributing to the poor reproducibility across current assays employed to evaluate Fc-mediated antibody effector functions.

## Introduction

Antibodies are commonly purified prior to analysis, and high throughput easy to use immunoaffinity formats for small-scale purification of IgG have become increasingly popular. This is especially the case where purification of small quantities of IgG from plasma are required for various assays and downstream analysis. Purified IgG is often studied in large cohorts and vaccine trials, where the efficiency of antibody-induced effector functions are evaluated and compared.

Diverse approaches exist for the purification of IgG from plasma, other biological fluids, and culture medium. These include ammonium sulfate precipitation, ion exchange chromatography, and affinity purification on immobilized Protein A and Protein G. A relatively recent, novel approach however is the use of Melon Gel, a proprietary resin chemistry and optimized buffer system (Thermo Fisher Scientific, USA). In contrast with positive selection methods such as Protein G and Protein A, Melon Gel binds all non-γ-globulin and plasma proteins while allowing purified IgG to be collected in the flow-through fraction. This method presents several potential advantages, in particular the absence of harsh low pH elution conditions commonly used during IgG immunoaffinity purification procedures such as Protein G. In contrast, Protein G affinity matrices for purification of IgG consist of a bacterial protein which primarily binds IgG at the at the Cγ2/Cγ3 interface (1), as well as to a low-affinity site in the Cγ1 domain (2). Several studies have highlighted potentially undesirable consequences of exposing IgG to low pH, and it has been suggested that Protein A and Protein G immunoaffinity methods which elute in the range of pH 2–3 should be reconsidered (3–7). This concern has arisen from findings that exposure to low pH results in partial denaturation, dramatic alteration of antigen-binding behavior, and conformational changes leading to aggregation (6–9). This concern is further highlighted by high-resolution NMR demonstrating that the second constant domain (Cγ2) of IgG collapses entirely at pH 3.1 (8) while remaining intact at pH 3.5 and above. What effect these widely used purification approaches have on functional activity is however, poorly understood. A small number of studies on the antigen binding region of IgG have demonstrated that low-pH IgG purification approaches dramatically alter F(ab')_2_ antigen recognition (5, 10, 11). Whether Fc dependent functions are also altered following IgG purification and low-pH elution, to our knowledge has not been investigated.

Several important functions of the immune system are associated with the Fc portion of IgG, such as binding to complement and Fcγ receptors (FcγRs). FcγRs play a central role in the immune system by connecting humoral and cell-based innate immunity, and the binding kinetics of IgG-FcγR interactions are key indicators of antibody functional performance. Crystal structures of the Fc fragment have proposed that FcγR bind asymmetrically to the top region of the Cγ2 domain, and the adjacent lower hinge of IgG (12, 13). The Cγ2/Cγ3 interface of the IgG Fc binds both Protein G and Protein A. Considering the aforementioned effects of low pH immunoaffinity methods, particularly the stability of the Cγ2 domain critical in FcγRs binding, it was hypothesized that exposure to low pH during Protein G purification might result in altered FcγR interactions, similar to the altered antigen recognition by the F(ab')2 region observed in other studies (5, 10, 11). Herein we compare the traditional Protein G immunoaffinity method of purifying IgG from plasma, with a method which elutes at physiological pH (Melon Gel). For both methods FcγR interactions were examined, and well-recognized factors impacting Fc tail functionality with the potential to significantly influence IgG binding; such as IgG subclass distribution, *N*-glycosylation, and aggregation, were compared.

## Materials and Methods

### Plasma Samples

Subsets of plasma samples used for this study were obtained from a previously published influenza vaccine cohort (14), consisting of 30 healthy HIV uninfected subjects, and 27 HIV positive subjects recently vaccinated against influenza. Ethics approval was provided by the Alfred Health and University of Melbourne Human Ethics Committees (IDs 432/14 and 1443420). All samples were collected at mean 28 days post vaccination with an intramuscular injection of trivalent influenza vaccine (Fluvax; bio-CSL, Australia) containing 15 ug of hemagglutinin (HA) from A/California/7/2009 (H1N1), A/South Australia/55/2014 (H3N2), and B/Phuket/3073/2013. A/South Australia/55/2014 is an A/Switzerland/9715293/2013 (H3N2)-like virus selected for inclusion in trivalent influenza vaccines for the Southern Hemisphere, with only a single K207R mutation (H3 numbering) differentiating these two antigenically similar strains. Thus A/Switzerland/9715293/2013 (H3N2) was used as a substitute HA antigen within this study.

### IgG Purification

IgG was purified from plasma samples from healthy individuals, or from HIV positive individuals (14), via Protein G chromatography (Protein G HP Multitrap, GE Healthcare, Sweden) and Melon Gel chromatography (Melon Gel IgG Purification Kit, Thermo Fisher Scientific, USA). For Protein G sepharose purification, plasma was diluted 2-fold with binding buffer (20 mM sodium phosphate, pH 7.0) and incubated for 1 h. IgG was eluted with 0.1 M glycine-HCl (pH 2.7), into neutralizing buffer (1 M Tris-HCl, pH 9.0). IgG was purified from plasma samples by Melon Gel purification according to the manufacturer's protocol. Briefly, serum samples were diluted 1:10 and the diluted serum was added to a minispin column containing the Melon Gel resin. After 5 min incubation, the purified IgG was collected in the flow through following the manufacturer's instructions. Eluted IgG (Protein G) and flow through (Melon Gel) were concentrated, and buffer exchanged into PBS using a 30 kDa Amicon Ultra centrifugal filter (Millipore, USA). IgG concentrations were quantitated using the Human-IgG ELISA kit (Mabtech, Sweden).

### Fcγ Receptor-Binding Kinetics of Protein G and Melon Gel Purified IgG

The kinetic constants of the interaction of Melon Gel and Protein G purified IgG and the low and high affinity variants of FcγRIIIa or FcγRIIa were determined by surface plasmon resonance BIACORE 3000; (GE Healthcare, Sweden). Biotinylated FcγRs >95% pure based on SDS–PAGE (SinoBiological Inc, China) were immobilized on a streptavidin sensor chip (GE Healthcare, Sweden) at a flow rate of 10 μl/min, with flow cell one left blank as a reference surface. Each FcγR was immobilized at the following ligand densities FcγRIIaH131: 270 RU, FcγRIIaR131: 917 RU, FcγRIIIa F158: 798 RU, and FcγRIIIaV158: 660 RU. To collect kinetic binding data, each analyte (purified IgG) in 10 mM HEPES, 150 mM NaCl, 0.005% P20, 0.1% Tween 20 pH 7.4, was injected over flow cells at concentrations of 333.3, 166.7, 83.3, 41.7, and 20.8 nM at a flow rate of 30 μl/min, and at a temperature of 25°C. For the FcγRIIIa ligands, the IgG was allowed to associate and dissociate for 120 and 360 s, respectively. The surfaces were regenerated with 2 × 5 μl injections of 10 mM Glycine HCl pH 2.25, for Protein G purified samples, and a single 5 μl injection of 10 mM Glycine HCl pH 3.0 for Melon Gel samples. For the FcγRIIa ligands, the complex was allowed to associate and dissociate for 120 and 360 s, respectively, and the surfaces were regenerated with 3 × 5 μl injections of 10 mM NaOH for Protein G, and a single 5 μl injection of 10 mM Glycine HCl pH 3.0 for Melon Gel samples. Data was double referenced and globally fitted to a 1:1 interaction model with Scrubber2 software (BioLogic Software, Australia).

### Real-Time Interaction Analysis of the Binding of IgG to FcγRIIa Exposed to Varying pH

Melon Gel purified IgG (9.5 mg/ml) (pooled from plasma of healthy individuals) was exposed to 0.1 M glycine buffer (low pH 1.5, to high pH 9.5) for 1 min. The pH was then adjusted to pH 7.0 and the samples were buffer exchanged into PBS. Binding to the higher affinity variant of FcγRIIa-H131 was determined by surface plasmon resonance on a BIACORE 3000 (GE Healthcare, Sweden). ~200RU FcγRIIaH131 was immobilized on a streptavidin sensor chip (GE Healthcare, Sweden). IgG samples were diluted in 10 mM HEPES, 150 mM NaCl, 0.005% P20 pH 7.4 and run over flow cells at a concentration of 333.3 nM at a flow rate of 30 μl/min. For the native sample, a 1 in 100 dilution of a plasma pool was used. The IgG complexes were allowed to associate and dissociate for 120 and 300 s, respectively.

### FcγR Dimer Receptor ELISA

An ELISA previously described (12, 15, 16) was used to characterize the FcγR potential functional capacity of IgG purified by Melon gel and Protein G from plasma. This assay employs dimeric recombinant soluble FcγR proteins (rsFcγR) to evaluate and model the cross-linking of antibody Fc with FcγRs as a measure of their potential to activate effector cells. Briefly, biotin tagged recombinant soluble homodimers of either FcγRIIIa or FcγRIIa were used to quantitate the FcγR-binding capacity of purified IgG from the subjects bound to influenza HA H3N2 (A/*Switzerland*/9715293/2013, Sinobiological Inc, China), or of HIV exposed individuals to HIV-1 _BAL_ gp120 (NIH-AIDS Reagent Repository, USA). The influenza or HIV antigens were prepared in PBS and adsorbed (50 ng/well) to 96 well plates (Nunc™ MaxiSorp™, Denmark) Wells were washed with PBS containing 0.05% Tween 20 and blocked with PBS containing 1 mM EDTA and 1% (w/v) BSA (Sigma-Aldrich, USA). Purified IgG samples were serially diluted from a starting concentration of 100–12.5 μg/ml, and then incubated for 1 h at 37°C. Plates were washed five times with PBS containing 0.05% Tween-20, and the Ab-bound plates were subsequently incubated with 0.1 μg/ml purified dimeric rsFcγRIIIa V158-biotin or 0.2 μg/ml purified dimeric rsFcγRIIa H131-biotin in PBS containing 1 mM EDTA, 0.05% Tween-20, and 1% (w/v) BSA for 1 h at 37°C. Following incubation plates were washed five times and High Sensitivity Streptavidin (Thermo Fisher Scientific, USA) 1/10,000 in diluent buffer was added for 1 h at 37°C. Plates were washed eight times and then developed with TMB Single Solution (Sigma-Aldrich, USA). The reaction was stopped by addition of 1 M HCl, and absorbance at 450 nm was determined. A standard curve was used to determine the geometric mean FcγR activating capacity of each IgG preparation, with standard curves generated with pooled human IgG: IVIg (Privigen®, CSL Behring, Australia), and HIV IgG (NIH-AIDS Reagent Repository, USA), respectively.

### Antibody Dependent Cellular Phagocytosis Assay (ADCP)

The THP-1 ADCP phagocytosis assay was performed as previously described (17). Briefly, Influenza HA protein H3N2 A/*Switzerland*/9715293/2013 (Sinobiological Inc, China), was biotinylated using the EZ-Link Sulfo-NHS-LC-Biotin kit (Thermo Fisher Scientific, USA) following manufacturer's recommendations. Biotinylated HA antigen was then incubated overnight at 4°C with fluorescent 1 μM neutravidin beads (Molecular Probes Inc, USA), and subsequently washed twice to remove unbound antigen. Purified IgG (10 μg/ml) was added to each well, and the plate was incubated for 2 h at 37°C in order to allow antibodies to bind to the beads. Controls with no IgG antibody, and with unconjugated beads were also run to determine background phagocytosis of the beads. 1 × 10^5^ THP-1 cells (monocytic cell line, ATCC TIB-202) were then added to each well, and the plate was incubated overnight at 37°C. Following incubation, cells were fixed in 1% formaldehyde before being acquired on a LSR Fortessa (BD Biosciences, USA) Flow cytometer. Flow cytometry data was analyzed using FlowJo analysis software version 10.5.3. Phagocytic scores were calculated as the geometric mean fluorescent intensity (MFI) of the beads multiplied by the percentage bead uptake. As a control to rule out any possible contaminating differentiating factors in the purified IgG samples, each sample was also preincubated with unconjugated beads, and any non-specific background for each sample was subtracted. Both the percentage of THP-1 cells that ingested one or more beads (percent bead positive), and the “mean phago score,” which was calculated by determining the percentage of cells that were bead-positive, and multiplying by the mean fluorescence intensity (to provide a convenient quantitative measure of net phagocytosis), was determined for each duplicate sample. For ease of presentation, these scores were then divided by 10^5^.

### Antibody Isotyping

Purified IgG or plasma samples were simultaneously quantified for IgG_1_, IgG_2_, IgG_3_, IgG_4_, IgA, and IgM using the Bio-Plex Pro™ Human Isotyping Panel, 6-plex kit (Bio-Rad, USA), according to the protocol provided by the manufacturer. Purified IgG and plasma samples were diluted 1/10,000 and 1/40,000, respectively. Plates were washed using a magnetic plate-washer (Bio-Plex Pro Wash station, Bio-rad, USA), read on a Bio-plex MAGPIX instrument, and analyzed on Bio-Plex Manager 6.1.1 (Bio-Rad, USA) software. The proportion of each IgG subclass was calculated as a total percentage of the sum of the concentration of all the four IgG subclasses.

### Mass Spectrometry of IgG *N*-glycans

#### SP3 Protein Clean Up and In-solution Digestion

Four Melon Gel and four matched Protein G IgG preparations (10 μg) were prepared for MS analysis using a modified SP3 sample preparation approach (18, 19). Briefly, samples were first denatured and reduced using 1% SDS, 10 mM DTT, 100 mM HEPES by boiling at 95°C, 1,000 rpm for 10 min. Samples were then cooled and alkylated with 40 mM 2-chloroacetamide (CAA) for 1 h at RT in the dark. The alkylation reactions were then quenched with 40 mM DTT for 10 min and then samples precipitated on to SeraMag Speed Beads (GE Healthcare, USA) with ethanol (final concentration 50% v/v). Samples were shaken for 10 min to allow complete precipitation onto beads, and then washed three times with 80% ethanol. The precipitated protein covered beads were then resuspended in 100 mM Ammonium Bicarbonate containing 1 μg of trypsin 1/10 (w/w) (Sigma-Aldrich, USA), and allowed to digest overnight at 37°C. Upon completion of the digests, samples were spun down at 14,000 g for 5 min to pellet the beads. The supernatant was then collected and desalted using homemade C18 stage tips (20) before being dried down and stored until analyzed by LC-MS. Prior to loading, samples were reconstituted in MS running buffer [2% acetonitrile (ACN), 0.1% trifluoroacetic acid (TFA)] to a concentration of 0.5 μg/μl of which 2 μg (4 μl) was loaded for analysis.

### iBAQ Proteome and Glycoform Analysis by LC-MS

Purified peptides were resuspended in Buffer A and separated using a two-column chromatography set up comprising a PepMap100 C18 20 mm × 75 μm trap and a PepMap C18 500 mm × 75 μm analytical column (ThermoFisher Scientific). Samples were concentrated onto the trap column at 5 μl/min for 6 min and infused into either an Orbitrap Elite™ Mass Spectrometer (ThermoFisher Scientific) for iBAQ based analysis or an Orbitrap Fusion™ Lumos™ Tribrid™ (ThermoFisher Scientific) for glycopeptide analysis at 300 nl/min via the analytical column using a Dionex Ultimate 3000 UPLC (ThermoFisher Scientific). Sixty-five minutes gradients were run altering the buffer composition from 1% buffer B to 28% B over 35 min, then from 28% B to 40% B over 10 min, then from 40% B to 100% B over 2 min, the composition was held at 100% B for 3 min, and then dropped to 3% B over 5 min and held at 3% B for another 10 min. The Orbitrap Elite™ Mass Spectrometer was operated in a data-dependent mode automatically switching between the acquisition of a single Orbitrap MS1 scan (120,000 resolution) followed by 20 data-dependent CID MS2 events (NCE 35) with 30 s dynamic exclusion enabled. The Orbitrap FusionTM LumosTM Mass Spectrometer was operated in a data-dependent mode automatically switching between the acquisition of a single FTMS MS1 scan (120,000 resolution) every 3 s and MS2 HCD fragmentation (NCE 30, a maximum injection time of 22 ms and AGC of 2 × 105) with 30 s dynamic exclusion enabled. If the HexNAc oxonium ion (m/z 204.087) and it associated ions m/z 186.075 and m/z 168.065 were detected within an HCD scan the putative glycopeptide precursor was subjected to addition characterization using ITMS CID (NCE 35) and FTMS EThcD (using charge dependent ETD reaction times, HCD NCE 25, a maximum injection time of 250 ms and AGC of 2 × 105).

#### Mass Spectrometry Data Analysis

For proteomic comparison of IgG preparations MS raw files were searched against the UNIPROT human proteome databases (UP000005640, downloaded July 2017) using MaxQuant [version 1.5.3.3 (21)]. Searches were performed using cysteine carbamidomethylation as a fixed modification, methionine oxidation and N-terminal acetylation as variable modifications with trypsin specificity and a maximum of two miss-cleavage events. The default MaxQuant instrument setting for Orbitrap FTMS (MS1 tolerance of ±20 ppm for the first search and ±4.5 ppm for the main search) and ITMS (MS1 tolerance of ±0.6 Da) were used with the resulting data filtered to a False discovery rates (FDR) of 1% at the protein and peptide levels. To enable the assessment of relative protein amount within IgG preparations the IBAQ (22) setting was enabled. The resulting output file was then imported into R for generation for data visualization.

The identification of IgG glycoforms within IgG preparations were accomplished using Byonic [Protein Metrics, version 3.4 (23)]. Each MS raw file was searched with a MS1 tolerance of ±10 ppm and a tolerance of ±20 ppm was allowed for both HCD and EThCD MS2 scans. Searches were performed using cysteine carbamidomethylation as a fixed modification, methionine oxidation as a variable modification in addition to allowing *N*-linked glycosylation on asparagine. The default Byonic human *N*-linked no multiple fucose glycan database, which is composed of 182 mammalian *N*-glycans compiled from literature sources (7–10), was used. The proteases specificity was set to full trypsin specificity and a maximum of two miss-cleavage events allowed. Data searched against the UNIPROT human proteome databases (UP000005640, downloaded July 2017). Search was filtered to a 1% protein FDR as set in the Byonic parameters with the final results collated using R with an additional filtering step to remove glycopeptide assignments with Byonic score below 150 [as suggested by Lee et al. (24)] applied to remove low quality glycopeptide assignments and ensure a <1% FDR.

Only glycopeptides of the main IgG subclass IgG_1_ were examined, as the other subclasses were confounded by the fact that in Caucasian populations the tryptic glycopeptide of IgG_3_ generally has the same peptide sequence as IgG_2_ (25), and IgG_4_ counts were poor for many of the glycopeptides. Fc IgG1 glycosylation at site N180 was compared across all samples. To compare glycosylation, peptide spectrum matches (PSM) i.e., the count of each time the glycopeptides were identified was quantified. To permit comparison across the samples, all samples were normalized to total count (sum of each time the glycopeptide is identified across the samples). Glycosylation features were calculated from the data: fucosylation (% of glycans bearing a core fucose), bisection (% of glycans with a bisecting GlcNAc), and sialylation (% of antennae carrying a sialic acid).

### Protein Aggregation Assay

A pool of Melon Gel purified IgG (9.5 mg/ml) was aggregated at 65°C for 30 min. At this point, the IgG was considered 100% aggregated (26). The aggregated IgG was then added in various proportions to unaggregated monomeric IgG to achieve percentages of aggregation, in order to create a standard curve as described by the manufacturer of 100, 80, 40, 20, 10.5, 2.5, and 0% aggregated IgG. Two microliters of ProteoStat® Fluorescent Dye (Enzo Life Sciences, USA) was added to each test sample containing 200 μg/ml of IgG in a 96-well black, clear flat-bottomed plate (Greiner Bio-One, Austria). This dye is widely used to detect protein aggregates (27) and is benchmarked with IgG by the manufacturer (28). The microplate containing test samples was incubated in the dark for 15 min at room temperature. Samples were excited at 530 nm and emission was read at 605 nm on a FLUOstar plate reader (BMG Labtech, Germany).

### ANS Fluorescence

Melon Gel or Protein G purified IgG samples were prepared at 2 μM and incubated with 32 μM of 8-Anilino-1-naphthalenesulfonic acid (ANS) (Sigma-Aldrich, USA) in a clear bottom, black, non-binding 96 well plate (Greiner Bio One, Austria). For endpoint measurement samples were excited at 388 nm and Emission at 500 nm was recorded. The fluorescence emission spectra of ANS was also recorded between 400 and 600 nm on a CLARIOstar plate reader (BMG Labtech, Germany).

### Statistical Analysis

Statistical analyses were performed using GraphPad Prism 8 (GraphPad Software, San Diego CA, USA). Data are summarized using descriptive measures such as median + IQR (interquartile range), mean ± SD, and percentage (%). Spearman's rank correlations were used to examine bivariate associations between variables. Wilcoxon matched pair signed rank tests were used to compare Melon Gel and Protein G data. The Friedman test with Dunn's multiple comparisons was used to compare data obtained from the IgG subclass analysis. In all cases *P*-values <0.05 were considered to indicate statistical significance. In figures, asterisks denote statistical significance (^*^p ≤ 0.05; ^**^p ≤ 0.01; ^***^p ≤ 0.001; ^****^p ≤ 0.0001; ns = not significant) with comparisons specified by connecting lines.

## Results

### Low-pH Exposure in Protein G Purified Samples Induces Aggregation of IgG

**Figure 1:**
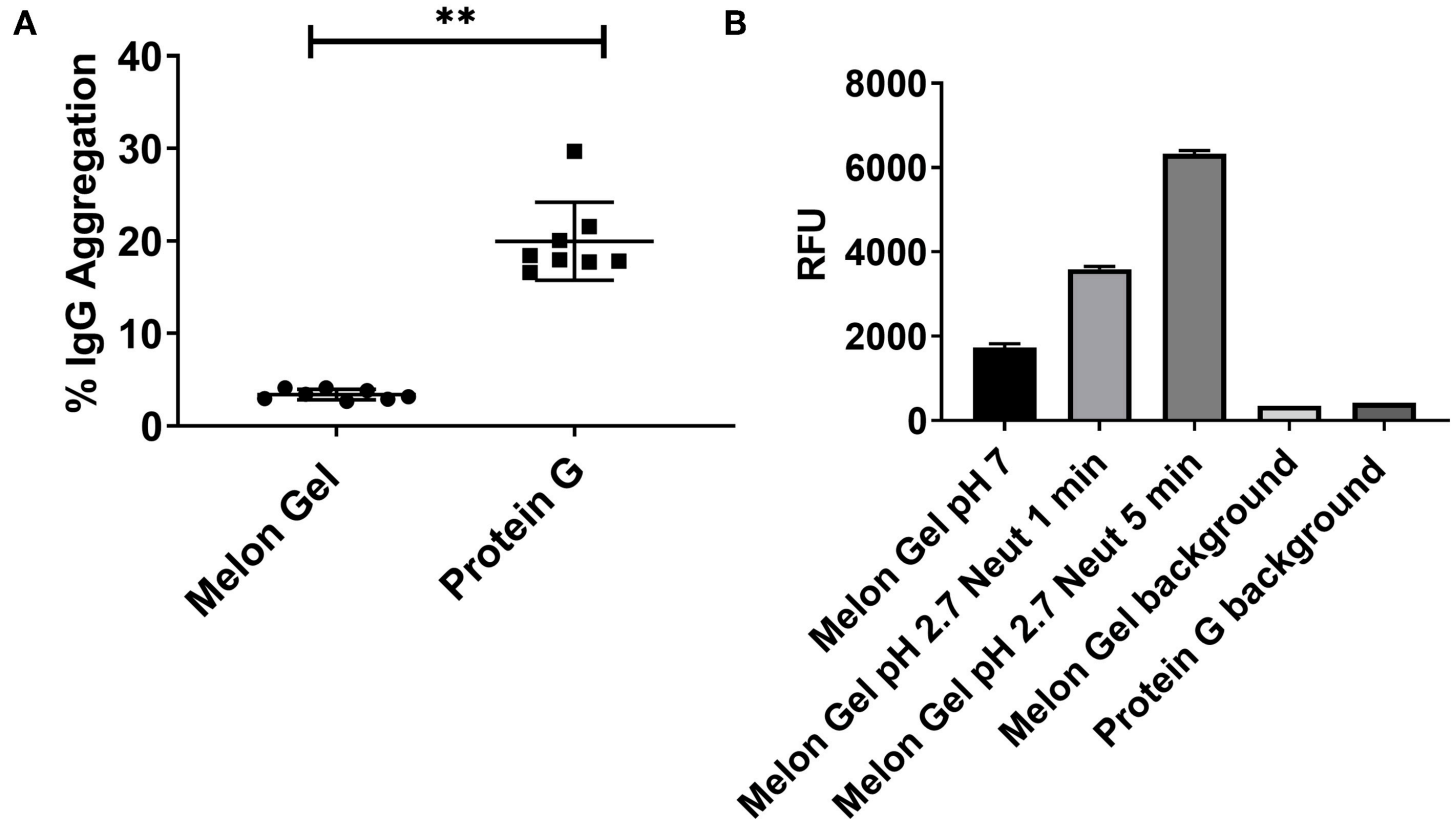
**(A)** Median + IQR IgG aggregation observed from a set of *n* = 8 matched paired samples of plasma IgG purified via Melon Gel and Protein G. **(B)** Increasing fluorescence with low pH exposure of a Melon Gel sample after 1 and 5 min. Mean ± SD of a of duplicate samples, exposed to Protein G elution buffer (0.1 M glycine-HCl, pH 2.7) and then neutralized with 1 M Tris-HCl, pH 9.0.

Potential differences in aggregation between Melon Gel and Protein G purified IgG were examined using a fluorescent dye, ProteoStat® (Enzo Life Sciences, USA). The fraction of aggregation in Melon Gel purified IgG from a subset of healthy serum samples (*n* = 8) was compared with that of equivalent IgG samples purified using Protein G. The Protein G purified samples were found to contain ~6-fold more aggregates than the Melon Gel purified IgG (Protein G median = 18.2 vs. Melon Gel median = 3.3, *p* = 0.0078; Figure 1A). In order to confirm the pH dependency of an aggregation affect, Melon Gel IgG was additionally exposed to the low pH elution buffer. When Melon Gel purified IgG was transiently exposed to the low-pH buffer conditions (pH 2.7) used in the elution of IgG in the Protein G purification process, the fluorescence of the ProteoStat® aggregate reporter dye almost doubled within a 1 min exposure and was increased 3-fold by 5 min (Figure 1B). The low pH elution step in the Protein G purification process is thus likely to strongly contribute to the higher aggregate content of IgG purified using Protein G.

### Low pH Exposure Increases IgG Hydrophobicity as Evaluated by 8-anilnonaphthalene-1-sulfonate (ANS) Fluorescence

**Figure 2:**
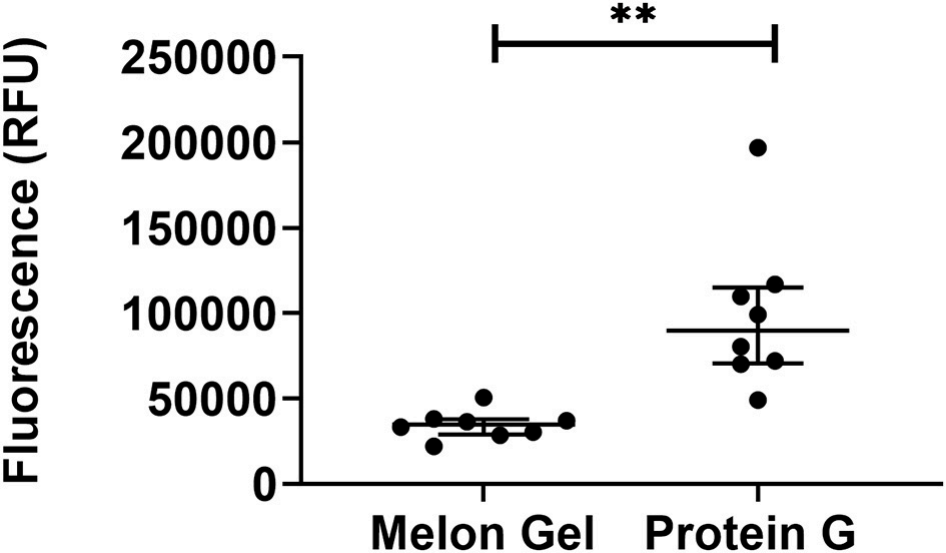
Low-pH buffer exposure of IgG results in an enhanced hydrophobic effect. Median + IQR fluorescence intensity of 32 μM ANS in the presence 2 μM of *n* = 8 matched purified Melon Gel and Protein G samples.

**Figure 3:**
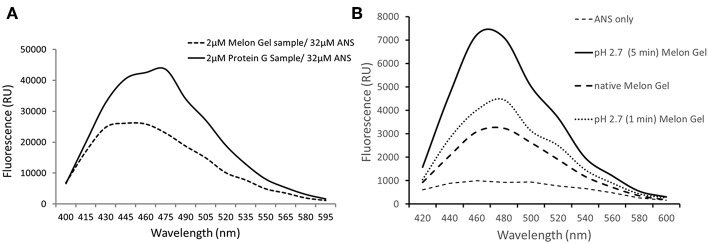
**(A)** Emission spectra of 32 μM ANS in the presence of 2 μM of a Protein G sample and a Melon Gel sample. **(B)** Emission spectra of 32 μM ANS in the presence of 2 μM of a native and pH 2.7 buffer exposed (5 min or 1 min) Melon gel sample.

ANS is a fluorescent molecular probe which can change its fluorescent properties as it binds to hydrophobic regions of proteins, thereby making it a useful tool to study conformational changes. ANS was therefore used as a probe to evaluate whether low-pH buffer exposure during the Protein G purification process exposed new hydrophobic sites on IgG. Protein G samples incubated in the presence of ANS exhibited a significantly higher fluorescence in comparison to Melon Gel purified IgG samples (Figure 2 *p* = 0.0078). Increased fluorescence intensity and a blue shift in the fluorescence maxima were also observed in the spectral scan recorded after excitation at 388 nm (Figure 3A), demonstrating the increased hydrophobicity of low-pH buffer-exposed IgG molecules. To confirm the low-pH dependency of this effect, a Melon Gel sample was also exposed to low pH for 1 and 5 min, and was also found to exhibit the same increased ANS fluorescence, consistent with an increased hydrophobicity upon exposure to low-pH (Figure 3B).

### Aberrant Kinetics of Protein G Purified IgG With Immobilized FcγRIIIa and FcγRIIa

**Figure 4:**
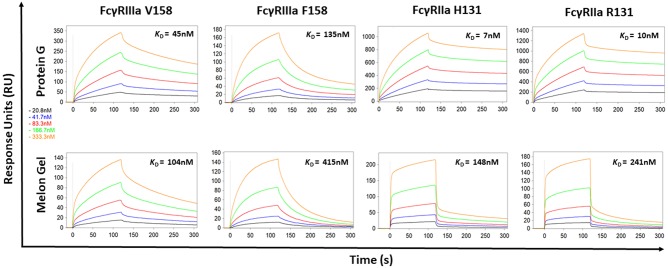
Binding kinetics of the high and low affinity variants of FcγRIIIa and FcγRIIa to IgG purified via Melon Gel and Protein G. Real-time surface plasmon resonance sensorgrams and affinity constants determined from SPR analysis representative of an individual polyclonal IgG sample serially diluted 2-fold from 333.3 nM.

**Figure 5:**
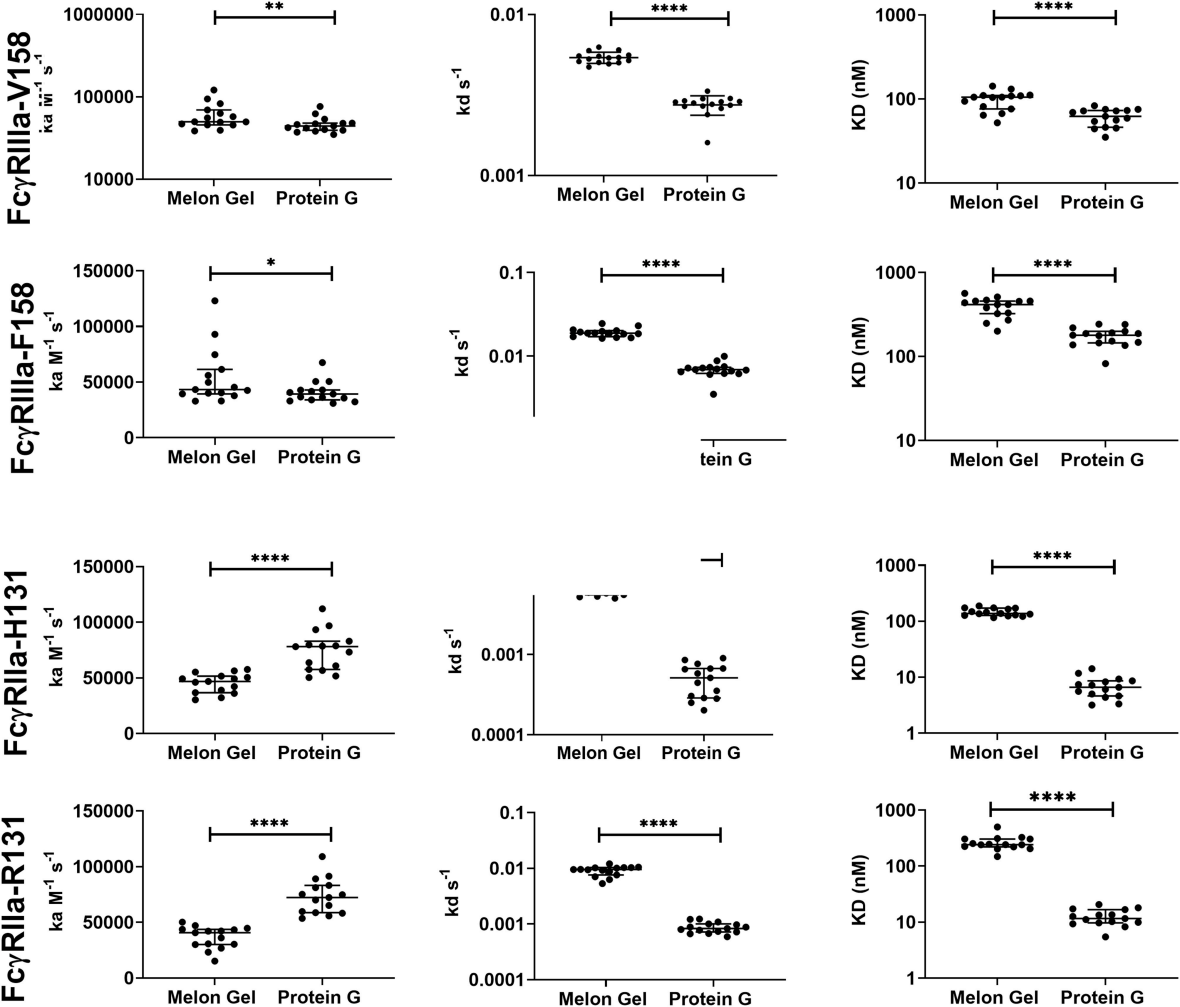
Kinetic constants (median + IQR) of a subset of *n* = 15 samples of polyclonal IgG purified from plasma either via Melon gel or Protein G immunochromatography. Kinetic constants were obtained by data globally fitted to a 1:1 interaction model. *p ≤ 0.05; **p ≤ 0.01; ****p ≤ 0.0001.

Protein G and Melon Gel purified IgG are widely used for the evaluation of antibody function, with many important functions mediated via Fc receptors. Whether the IgG purification method employed had any effect on the binding of purified IgG to Fc receptors was examined using surface plasmon resonance (SPR). The different Fc binding ectodomains of the allelic variants of FcγRIIIa and FcγRIIa were immobilized and reacted with either Protein G or Melon Gel purified IgG. Inspection of representative sensograms (Figure 4) and the apparent binding constants derived from the analysis of *n* = 15 IgG samples (Figure 5), found stronger binding by the Protein G purified samples. Most striking was Protein G purified IgG binding profile to immobilized FcγRIIa, where the bound analyte dissociated more slowly and the apparent k_D_^app^ obtained was about 20-fold lower than that of the Melon gel purified IgG (*p* < 0.0001). Notably, even the Melon Gel purified IgG showed biphasic binding profiles to immobilized FcγRIIa, that is characteristic of multivalent binding of aggregates but with a much smaller slow association and faster dissociation component than the Protein G-IgG. Nevertheless, even the low 3.3% level of aggregates in the Melon Gel purified IgG affected the binding sensograms and results in a higher apparent binding strength (FcγRIIa H131 K_D_^app^ = 0.14 μM and R131 K_D_^app^ = 0.24 μM) when fitted to a 1:1 binding model, than the known micromolar binding of this receptor (13, 29). FcγRIIIa has higher affinity for IgG-Fc than FcγRIIa but also showed divergent binding sensograms for Protein G and Melon gel purified IgG, although this was less extreme than for FcγRIIa. The K_D_^app^ of the Protein G IgG was over 2-fold lower (*p* < 0.0001), again consistent with the higher level of aggregates in this preparation contributing to stronger apparent binding to FcγRIIIa.

### IgG Exposure to Low-pH Produces Aberrant Binding Kinetics With Immobilized FcγRIIa

**Figure 6:**
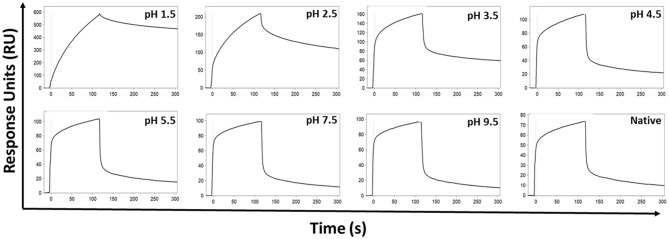
SPR real-time interaction analysis of the binding of 333.3 nM IgG to FcγRIIa. IgG was exposed transiently over a range of low to high pH of 0.1 M glycine-HCl, and then neutralized with 1 M Tris-HCl, pH 9.0. Native IgG (1:100 dilution of plasma) was also run for comparison.

The effect of the low pH elution buffer used in the Protein G purification process was evaluated by exposing IgG to the low-pH elution buffer, and the pH dependency of the binding to FcγRIIa was investigated. Here the binding profiles obtained after single injections of a pool of IgG purified via Melon gel transiently exposed to a range of low to high pH buffer were compared. Transient exposure of IgG at a pH of 1.5 or 2.5 strongly increased the binding of IgG to FcγRIIa, as evident by comparing the dissociation curve of the interaction to a pH of 3.5 and above (Figure 6). Pooled Melon gel IgG was also exposed to the binding buffer used in the Protein G purification procedure (20 mM sodium phosphate, pH 7.0), displaying a similar profile to that observed here at pH 7.5 (Figure S1).

### IgG Aggregates in Protein G Preparations Show Increased Dimeric FcγR Binding

**Figure 7:**
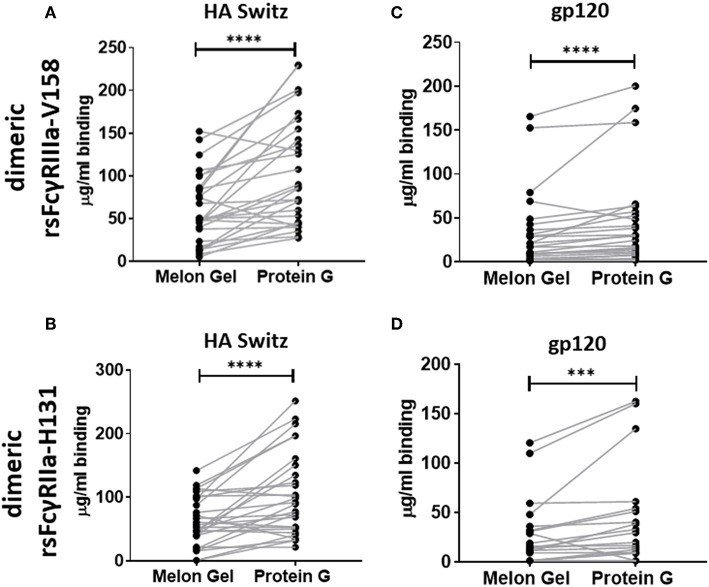
Dimer ELISA binding Ab responses to HA from A/Switzerland/9715293/2013 (H3N2) (HA Switz) for FcγRIIIa V158 *n* = 28 **(A)** and FcγRIIa H131 **(B)** and HIV gp120 protein **(C,D)** *n* = 25. IgG samples were serially diluted 4-fold from a starting concentration of 100 μg/ml. Melon Gel and Protein G responses were compared using the Wilcoxon matched-pair test. ***p ≤ 0.001; ****p ≤ 0.0001.

Dimeric rsFcγR ectodomains have been used as a serological approach to evaluate the functional, FcγR-activating, capacity of IgG-immune complexes. The binding of dimeric rsFcγR ectodomains in an ELISA requires avid binding to pairs of IgG-Fcs. Having found by SPR that aggregates in Protein G purified IgG bind avidly to FcγRs, the dimeric rsFcγR assay was employed to predict if these aggregates might also have FcγR activating capacity. Recombinant soluble biotin tagged homodimers of either FcγRIIIa or FcγRIIa high affinity variants were used to quantitate and compare the activating potential of IgG purified using the two distinct purification methods. IgG from 28 healthy individuals purified via Protein G and Melon Gel were incubated with Influenza H3 Switzerland hemagglutinin (HA) immobilized to ELSA plates and the capacity of these antigen immune complexes to bind dimeric rsFcγRs was measured. Increased binding to the Protein G IgG-immune complexes was observed for both dimeric rsFcγRIIIa and dimeric rsFcγRIIa (*p* < 0.0001, Figures 7A,B). Thus, HA-specific IgG in the Protein G preparations include some aggregated material which retains HA binding activity, and by virtue of aggregation, artefactually elevates binding avidity to FcγRs. This observation was extended using a second separate cohort consisting of plasma samples from 25 HIV positive individuals, from which polyclonal IgGs, were also purified using both methods and reacted with HIV gp120 antigen. Again dimeric rsFcγRIIIa (*p* < 0.0001) and dimeric rsFcγRIIa (*p* = 0.0006) rsFcγR binding in the ELISA was higher for Protein G purified samples (Figures 7C,D). This increased binding for dimeric FcγRIIIa and FcγRIIa suggests that Protein G IgG purified samples have augmented capacity to activate FcγR effector cells.

### Protein G Purified IgG Has Apparently Enhanced Effector Function

**Figure 8:**
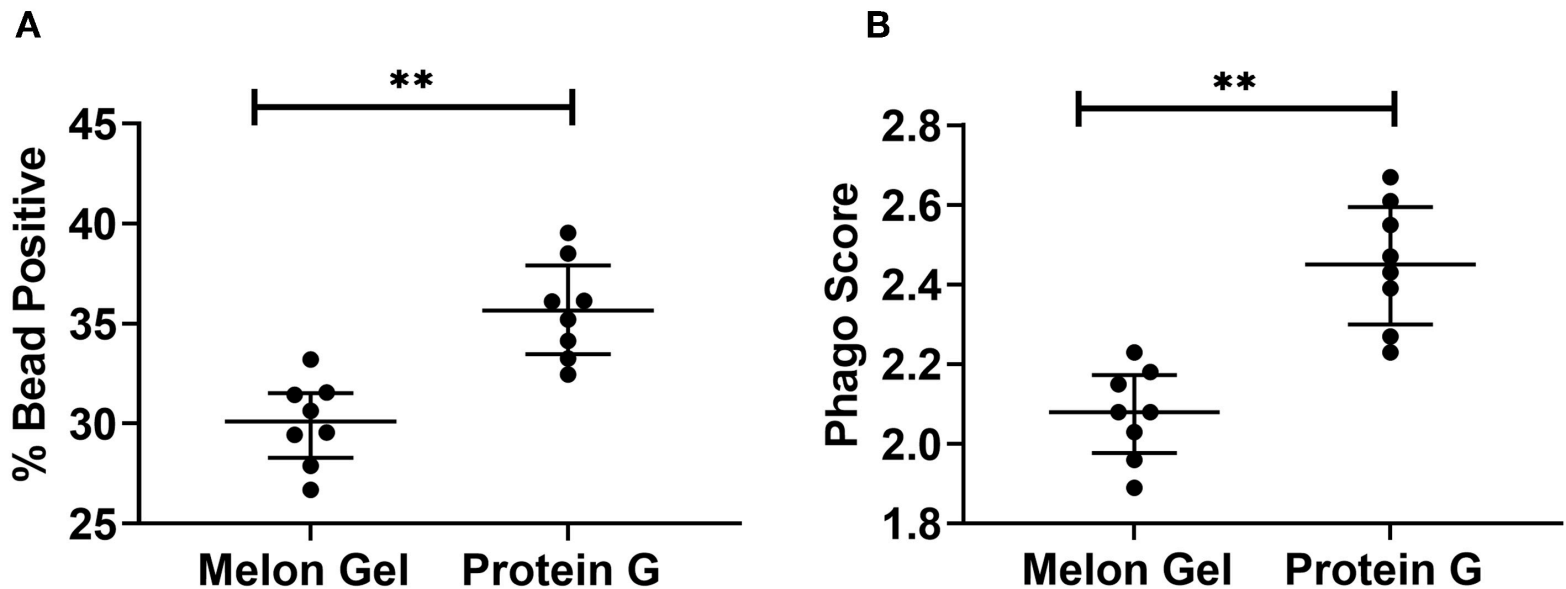
**(A)** Median + IQR % bead positive THP-1 cells, and **(B)** phagocytic scores of purified Protein G or Melon Gel IgG from flu vaccinated individuals against hemagglutinin-coated beads. All samples were run in duplicate, and Melon Gel and Protein G responses were compared using the Wilcoxon matched-pair test.

Next, we evaluated whether the aggregate-mediated increased avidity of Protein G purified IgG interactions with FcγRs translates to enhanced FcγR function in a well-validated *in vitro* phagocytosis cell-based assay. THP-1 human monocytic cells which express FcγRI and FcγRIIa were incubated with influenza HA Switz conjugated fluorescent beads that were opsonized with IgG from Influenza vaccinated individuals purified using either Melon Gel or Protein G. Phagocytosis of HA-beads by THP-1 cells (Figure 8) was significantly augmented when opsonized with Protein G purified IgG (Figure 8A % bead positive median = 35.7%; *p* = 0.008; Figure 8B Phagocytosis score *p* = 0.008; median = 2.45), compared to identical matched samples purified via Melon Gel (% bead positive median = 30.1%, Phagocytosis score median = 2.08). The MFI (representing the mean quantity of internalized antigen conjugated fluorescent beads per THP-1), was also observed to be significantly (*p* = 0.0078) higher in Protein G samples (Protein G median = 6,203 vs. Melon Gel median = 5,707, data not shown). Thus, antibody dependent cellular phagocytosis (ADCP) activity is artefactually increased upon purification using Protein G as are potentially other effector functions that depend on avid binding to FcγRs.

### Effect of Purification Method on IgG Subclass Distribution and Fc *N*-glycan Composition

**Figure 9:**
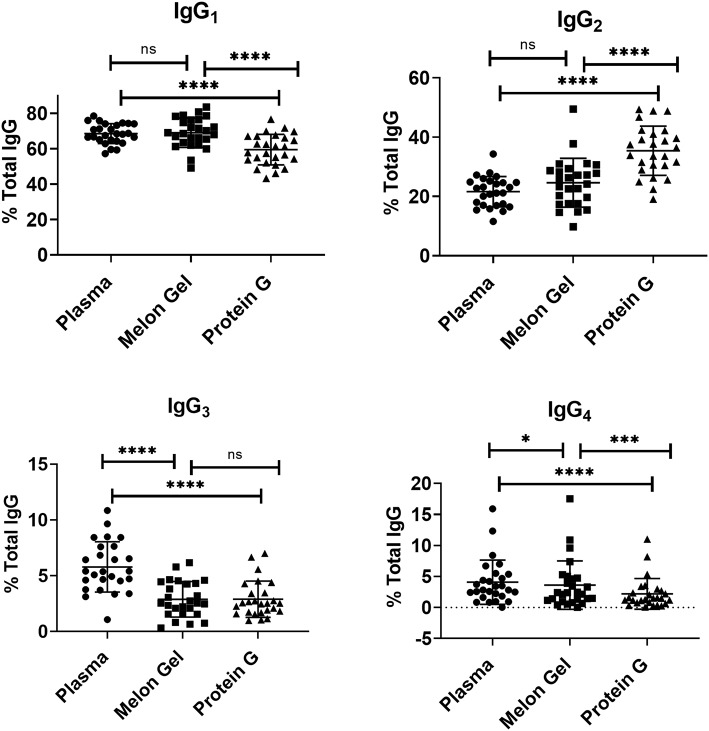
Mean and SD of subclass percentage obtained for plasma and purified (Melon Gel and Protein G) IgG samples (*n* = 26) via Multiplex immunoassay. The Friedman test with Dunn's multiple comparisons was used to compare data from each group. *p ≤ 0.05; ***p ≤ 0.001; ****p ≤ 0.0001.

Since IgG interactions with FcγRs are influenced by IgG subclass and glycosylation (30, 31), it is possible some attributes of the augmented activity of the Protein G purified polyclonal IgG may be attributed to altering the subclass or glycan composition. First, the IgG subclass composition of Protein G and Melon gel preparations was examined. Although it is known that Protein G binds all human IgG subclasses, there is no published data as to whether either Protein G or Melon Gel purification alters the subclass composition of plasma IgG. Matched Melon gel and Protein G purified IgG from healthy donors, along with corresponding plasma samples were simultaneously quantified for IgG_1_, IgG_2_, IgG_3_, and IgG_4_ via multiplex immunoassay, in order to determine which purification approach best reflected the IgG subclass distribution ratios observed in plasma (Figure 9). Compared to the subclass composition of plasma both the Melon Gel purified samples and Protein G purified IgG samples had lower levels of IgG_3_ (*p* < 0.0001). A small deficit in the proportion of IgG4 in Melon gel preps contrasted with the Protein G preparations which had substantially decreased proportions of IgG1, IgG3, and IgG4, while IgG2 was overrepresented (*p* < 0.0001). The FcγRIIa-H131 allele is the only human FcγR that functionally binds human IgG2. Thus, although Protein G preparations had increased proportions of IgG2, the enhanced binding of the low-IgG2 reactive FcγRIIa R131 allele with these preparations by SPR (Figures 4, 5) and dimeric receptor assay (Figure 7) indicates this enhanced activity is mediated, not by the higher IgG2 composition, but by the higher aggregate composition in the Protein G preparations. However, these alterations in subclass composition, including increased IgG2, could have important effects in other functional evaluations. Nevertheless, both methods showed a significant correlation, both to each other and to plasma with regard to IgG subclass distribution (Table S1).

**Figure 10:**
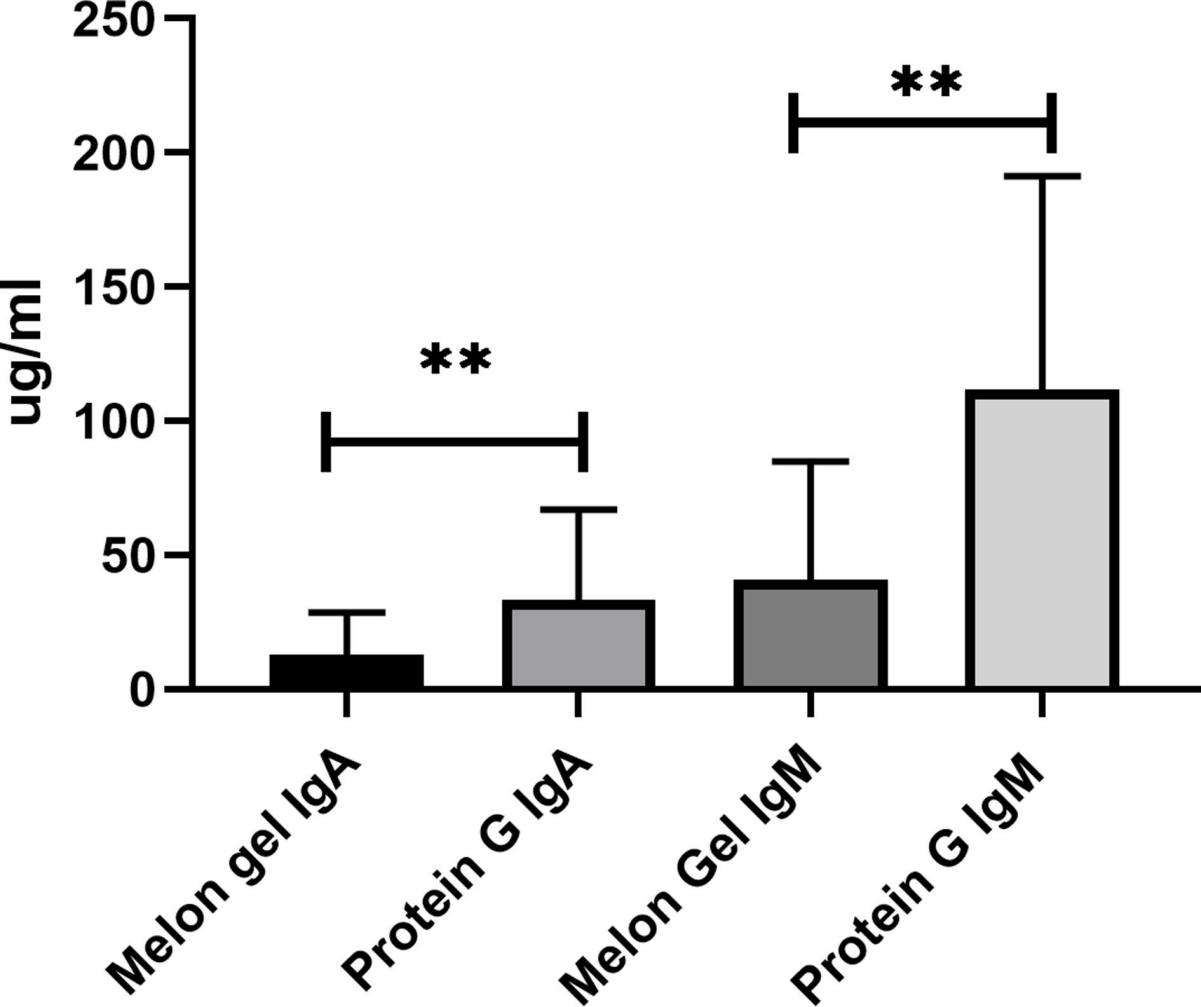
Median + IQR of concentration of non-IgG immunoglobulins (IgA and IgM) observed in Melon Gel vs. Protein G purification, *n* = 8.

Compared to antibody affinity purification with Protein A or Protein G, Melon Gel IgG Purification is reported to provide greater yields and higher purity by the manufacturer. Here, we also examined via multiplex immunoassay a subset of samples for the presence of contaminating immunoglobulins IgA and IgM by the two IgG purification approaches (Figure 10), with Protein G purified samples found to contain significantly more contaminating IgA (*p* = 0.0078) and IgM (*p* = 0.0078), equating to an almost 3-fold difference for both IgA (median = 12.9 vs. 33.3 μg/ml) and IgM (median = 41.0 vs. 111.9 μg/ml). Since the composition of asparagine (*N*) 297-linked glycans are well-known to modulate the binding affinity of IgG Fc to Fcγ receptors, an exploratory analysis using mass spectrometry was performed on four matched IgG samples purified via Melon Gel and Protein G. Fc IgG1 glycosylation at site Asn 297 (N180) was compared across all samples, however no differences were observed in this preliminary analysis (Figure S2).

## Discussion

Fc-functional antibodies are of growing interest in protection and control of diverse infectious diseases, such as HIV, Influenza, and Mycobacterium Tuberculosis (12, 30, 32, 33). Given that neutralizing antibody titers do not always correlate with protection, Fc-functional antibody assays are fundamental components of correlates of protection for the evaluation of these infectious disease research cohorts, as well as current and future vaccine efficacy trials (32–36). Quantitative functional antibody assays have however been reported to be difficult to standardize, and reliable assays that can be reproducibly used across different laboratories to measure Fc-dependent functions, such as antibody dependent cellular cytotoxicity (ADCC), have been described as limited (37–39). Consequently, there is a lack of consensus as to which Fc effector functions contribute to protection against a number of infectious diseases, such as HIV-1 (37, 39). Although a range of factors may be responsible for these for conflicting results, how the antibodies used in these functional assays have been purified, appears to be a technical factor which has been entirely overlooked. Given the wide array of diverse approaches that are available for researchers to purify IgG, it is reasonable to assume that the purification strategy employed, might also contribute to divergent results obtained across laboratories with respect to functional assays. Herein we compared two popular approaches for purifying IgG from human plasma and demonstrated that the method employed to purify IgG dramatically altered FcγR-binding behavior and Fc functional activity.

Typical purification processes which elute from affinity resins at low pH such as Protein A and Protein G, have been well-documented to promote formation of non-native IgG structures, particularly aggregates (40). Low-pH exposure of IgG has been found to be caused by Cγ2 unfolding associated with protonation of specific acidic residues, with IgG aggregation primarily determined by IgG subclass, and degree of Cγ2 glycosylation (8). As demonstrated in the present study, such aggregates show enhanced avid binding to Fc receptors, thus significantly impacting antibody binding kinetics and apparent affinity. Low pH exposure in our study resulted in 6-fold higher median percentage of IgG aggregates in the Protein G samples compared to those purified via Melon Gel. Furthermore, even Melon Gel purified IgG with only 3% aggregation appeared to be influenced by these aggregates in the SPR sensograms, with increased apparent binding affinity and decreased dissociation. Similar results were also obtained by Dorion-Thibaudeau et al. where even small percentages (2%) of IgG aggregates were found to affect Fc receptor binding (41). A previous study which investigated the effect of IgG aggregates on Fc receptor binding, also found aggregates bind more strongly to Fc receptors, with a much slower off-rate, significantly impacting affinity determinations (42). This is the result of an increased avidity effect, rather than a true difference in IgG affinity. Significantly slower apparent off-rates (*k*_d_) as observed via SPR were also a feature of Protein G purified samples in the present study. Indeed, in our study, the presence of aggregates in these low pH exposed Protein G samples was found to have a more profound effect on the low affinity FcγRIIa, where a 20-fold increase in apparent binding affinity was observed. The effect of aggregates for low affinity receptors, where the avidity effect would be more pronounced, has also been previously found to be much greater than that for high affinity receptors (43). The increased avidity of binding of Protein G purified IgG translated to apparently augmented effector function in our study, with Protein G purified IgG having an enhanced capacity to stimulate FcγRs on THP-1 cells to trigger ADCP. Future studies exploring the effect of low pH purification methods upon other IgG Fc-mediated functions such as antibody mediated complement activation is warranted.

Together SPR and dimer ELISA analysis data revealed a profound impact on the binding kinetics of Protein G purified IgG binding FcγRIIIa and FcγRIIa. SPR which is widely used to examine the binding activities of different FcγRs (29), is sensitive enough to detect composition changes, such as the increased presence of dimers and aggregates in IgG samples. The increased avidity, and vast difference in the apparent binding kinetics of Protein G and Melon Gel purified IgG was further highlighted in our results by the need to modify the regeneration conditions necessary to dissociate the ligand-analyte interaction for all Protein G purified IgG samples described in the SPR methods. We also observed significantly enhanced binding to specific antigens (HIV gp120, and influenza HA) via a previously described dimer ELISA, demonstrating that aggregates also preserve their antigen binding activity.

By using ANS, a fluorescent molecular probe used to examine hydrophobicity of proteins, we confirmed that exposure of IgG to low-pH results in molecular modifications characterized by a significant increase in hydrophobicity as observed by a previous study (5). The authors of this study theorized that low-pH triggers the exposure of previously buried hydrophobic amino acids, thereby increasing the hydrophobicity of IgG. The fact that we were able to induce changes to Melon Gel IgG by transient exposure to the low pH elution buffer used in the Protein G purification process, suggests that it is most likely low pH and not any other aspect of the Protein G purification procedure that is responsible for these conformational changes. Where on the IgG molecule these hydrophobic changes occur, and how exactly they trigger IgG aggregation, or whether they alter Fc receptor binding remains to be determined. The authors of the previous study assumed that these conformational changes occur in the Fab region, as the objective of their study sought to investigate the phenomenon of low pH induced antigen polyreactivity (5). One study, published over 50 years ago, however demonstrated by infrared spectroscopy as well as by hydrogen-deuterium exchange measurements that at low pH, the original structure of the IgG Fc fragment is altered, whereas the conformational properties of the Fab fragments do not change significantly (44). A study 4 years later (45) also supported these findings suggesting the refractory nature of the Fab region. Our findings demonstrate that a significant increased apparent binding affinity is observed when IgG is exposed to a pH below 3.5. Whereas, from a pH of 3.5 upwards, binding was found to reflect a similar profile to native IgG. This observation is interestingly consistent with high-resolution NMR analysis demonstrating that the second constant domain (Cγ2) of IgG remains intact at pH 3.5 and is profoundly altered at pH 3.1 (8). It is therefore plausible that low pH induced conformational changes to the Cγ2 domain occur, in addition to the increased presence of IgG aggregates, however this remains to be determined. Investigating the relative contributions of low pH induced IgG aggregation in comparison to low pH induce protein hydrophobicity upon increased apparent FcγR binding and functions may provide further insights.

It is well-known that the binding affinity of an IgG for Fc receptors can be modulated by IgG subclass (46), and each of the IgG subclasses has a unique binding profile to each FcγR. For the low affinity receptors FcγRIIa and FcγRIIIa examined in this study, binding affinity generally follows the hierarchy IgG_3_ > IgG_1_ >> IgG_2_ = IgG_4_, however, the allelic variant of FcγRIIa with a histidine at position 131 (H131) displays a higher affinity for IgG_2_ and has the binding hierarchy, IgG_3_ > IgG_1_~ IgG_2_ > IgG_4_ (13). For both FcγRIIa and FcγRIIIa binding affinity is strongest for IgG_3_, which did not differ significantly from the two purification methods. Furthermore, IgG_1_, which follows IgG_3_ in binding affinity, was significantly higher in the Melon Gel samples. It is therefore unlikely that absolute differences in IgG subclass ratios between the two purification methods contribute to the disparate binding profiles of IgG observed with the two purification methods. In particular the higher proportion of IgG2 in Protein G purified IgG did not directly enhance FcγRIIa binding as this was enhanced to both the H131 allelic form and the low-IgG2 binding R131 allelic form. It is relevant to note however, that IgG_2_ has been found to exhibit greater aggregation under low-pH conditions than IgG_1_ (8, 47). The greater proportion of IgG_2_ observed in the Protein G/low pH purified samples may consequently promote aggregation that incorporates universally FcγR binding IgG1 and IgG3 subclasses in mixed aggregates.

Glycosylation of the Fc region of IgG is well-known to be critical for maintaining IgG structural integrity, and modulating Fc receptor binding (48). It has been reported that sialic acids may be lost when they are subjected to high temperature (>28°C) or extreme pH with prolonged exposure, possibly resulting in the formation of false glycans in the sample (49). Our analysis was limited to glycopeptides of the main IgG subclass IgG_1_, as the other subclasses were confounded by the fact that in Caucasian populations the tryptic glycopeptide of IgG_3_ generally has the same peptide sequence as IgG_2_ (25), and IgG_4_ counts were poor for many of the glycopeptides. Though our preliminary limited exploratory analysis of four samples examining Fc IgG_1_ glycosylation analysis revealed no significant differences, this finding is not definitive, and whether diverse purification approaches impact Fc IgG glycosylation is a factor which warrants further investigation.

Much of the understanding surrounding IgG purification approaches, and IgG aggregation is known from biopharmaceutical antibody production (50) but may be less appreciated in the research community. Commercial monoclonal therapeutics are also often susceptible to aggregation during routine purification steps. For example, Nivolumab, an anti-programmed death (PD)1 IgG_4_ antibody used as a cancer treatment was found to be significantly affected by low pH Fc induced aggregation during routine Protein A chromatography (9). This is especially important as aggregates, and polyreactive IgG can threaten safety and efficacy by eliciting undesirable immunogenic responses. As such, many mAb therapeutics have moved away from low pH elution chromatography approaches, and modifications in the manufacturing process have been introduced to either minimize or remove aggregates in the final product (50). A positive aspect of exposing IgG to low pH has however been the enhancement of the therapeutic potential of immunoglobulin preparations such as IVIg in experimental systems (11). For example, low pH exposed, and not native treated intravenous immunoglobulin was found to improve the survival of mice with bacterial lipopolysaccharide-induced septic shock (5). The mechanisms behind this phenomenon have however been assumed to be attributed to structural modifications in the Fab region and the formation of aggregates, while the impact on Fc functionality has to our knowledge never been explored.

## Conclusion

Herein we demonstrate that exposing IgG to low pH significantly affected Fc-binding behavior and Fc effector functions as a result of IgG aggregation. Taken together with previous studies of monoclonal and polyclonal IgG purified by low pH elution from Protein G and Protein A, these findings indicate that transient exposure of IgG to low pH (<3.5), leads to exposure of hydrophobic sites and significant aggregation, whilst preserving antigen and FcγR binding. This results in apparently enhanced functionality in the context of binding and cell activation assays that are sensitive to the avidity of interactions. Since the normal function of the low affinity FcγRs is the avid sensing of immune complexes and antibody opsonized targets, the unappreciated impact of aggregates on evaluating antibody effector functions is evident in the present study, though the potential impact on other major FcγR receptors remains to be determined. These observations contribute to the mounting evidence highlighting the potential undesirable consequences of purification processes which elute from affinity resins at low pH, such as Protein G and Protein A, and suggest that IgG cannot be assumed to be fully native following low-pH elution. Thus, the purification strategy chosen by researchers may profoundly influence the outcome and interpretation of experimental systems, and may consequently be a previously overlooked factor contributing to the current lack of reproducibility between assays employed to evaluate Fc-mediated effector functions. Researchers should actively consider antibody purification methods both upon design of experiments and in the interpretation of experimental data.

## Data Availability Statement

The datasets generated for this study are available on request to the corresponding author.

## Ethics Statement

The studies involving human participants were reviewed and approved by Alfred Health and University of Melbourne Human Ethics Committees (IDs 432/14 and 1443420). The patients/participants provided their written informed consent to participate in this study.

## Author Contributions

EL wrote the manuscript, conceived, designed and performed all experiments, with the exception of the mass spectrometry glycan analysis which was performed and analyzed by NS. Recombinant soluble Fc Receptors were provided by PH and BW. Plasma samples were provided by AW and SK. AC and SK contributed to the design of the research and together with BW interpreted the results. All authors reviewed and contributed to the final manuscript.

### Conflict of Interest

The authors declare that the research was conducted in the absence of any commercial or financial relationships that could be construed as a potential conflict of interest.
