# Supplementary material for: Low pH Exposure During Immunoglobulin G Purification Methods Results in Aggregates That Avidly Bind Fcγ Receptors: Implications for Measuring Fc Dependent Antibody Functions
Source: Front Immunol. 2019 Oct 11;10:2415. doi: 10.3389/fimmu.2019.02415 (PMC6797627; doi:10.3389/fimmu.2019.02415)
Supplement: Supplementary file 1 [file Data_Sheet_1.docx]

| **Subclass** | **Comparison** | **r-Spearman** | **p-value** |
| --- | --- | --- | --- |
| IgG1 | Melon Gel and Protein G | 0.86 | <0.0001 |
|  | Melon gel and Plasma | 0.86 | <0.0001 |
|  | Protein G and Plasma | 0.88 | <0.0001 |
|  |  |  |  |
| IgG2 | Melon Gel and Protein G | 0.78 | <0.0001 |
|  | Melon gel and Plasma | 0.86 | <0.0001 |
|  | Protein G and Plasma | 0.82 | <0.0001 |
|  |  |  |  |
|  |  |  |  |
| IgG3 | Melon Gel and Protein G | 0.84 | <0.0001 |
|  | Melon gel and Plasma | 0.81 | <0.0001 |
|  | Protein G and Plasma | 0.93 | <0.0001 |
|  |  |  |  |
|  |  |  |  |
|  |  |  |  |
| IgG4 | Melon Gel and Protein G | 0.95 | <0.0001 |
|  | Melon gel and Plasma | 0.97 | <0.0001 |
|  | Protein G and Plasma | 0.97 | <0.0001 |

Table S1. Correlations between each of the IgG subclasses and Plasma obtained via Melon Gel and Protein G purification methods


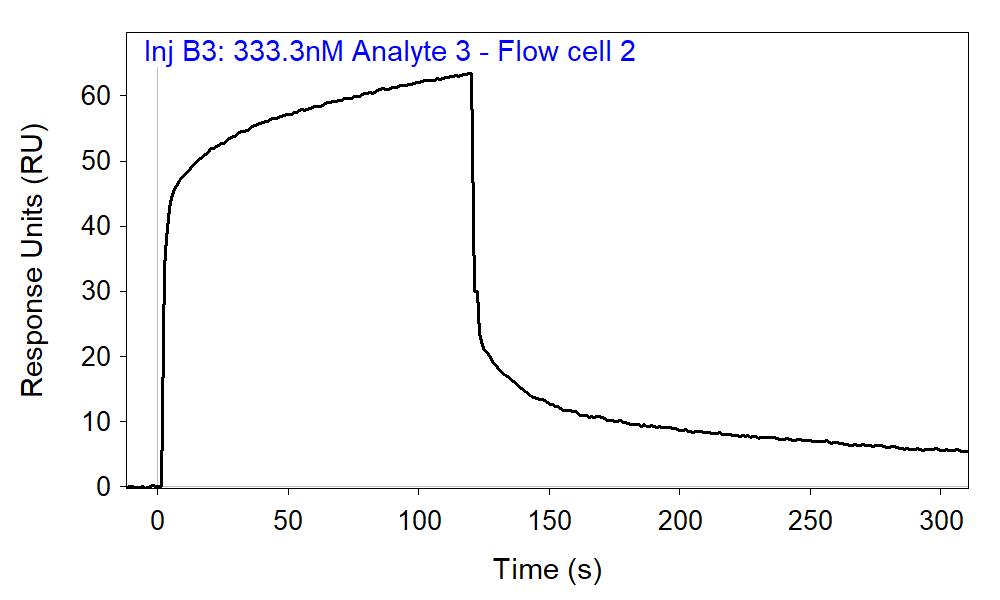


Figure S1. SPR real-time interaction analysis of the binding of 333.3nM IgG to FcγRIIa exposed transiently to Protein G binding buffer (20 mM sodium phosphate, pH 7.0)

Figure S2. Sum of Peptide spectrum matches (PSM) for each calculated glycosylation feature. Bisection (% of glycans with a bisecting GlcNAc), sialylation (% of antennae carrying a sialic acid), fucosylation (% of glycans bearing a core fucose). *w/o denotes without.

Glycoforms identified and included in the analysis are indicated below.

| HexNAc(1)Fuc(1) | - |
| --- | --- |
| HexNAc(2)Fuc(1) | - |
| HexNAc(3)Hex(3)Fuc(1) | - |
| HexNAc(3)Hex(4)Fuc(1) | - |
| HexNAc(4)Hex(3) | G0 |
| HexNAc(4)Hex(3)Fuc(1) | G0F |
| HexNAc(4)Hex(4) | G1 |
| HexNAc(4)Hex(4)Fuc(1) | G1F |
| HexNAc(4)Hex(4)Fuc(1)NeuAc(1) | G1S1F |
| HexNAc(4)Hex(5) | G2 |
| HexNAc(4)Hex(5)Fuc(1) | G2F |
| HexNAc(4)Hex(5)Fuc(1)NeuAc(1) | G2S1F |
| HexNAc(4)Hex(5)NeuAc(1) | G2S1 |
| HexNAc(5)Hex(3)Fuc(1) | G0FB |
| HexNAc(5)Hex(4)Fuc(1) | G1FB |
| HexNAc(5)Hex(4)Fuc(1)NeuAc(1) | G1S1FB |
| HexNAc(5)Hex(5)Fuc(1) | G2FB |
